# Therapeutic effects of curcumin on constipation-predominant irritable bowel syndrome is associated with modulating gut microbiota and neurotransmitters

**DOI:** 10.3389/fmicb.2023.1274559

**Published:** 2023-12-14

**Authors:** Xiaoting Tu, Hongyan Ren, Shurui Bu

**Affiliations:** ^1^Department of Gastroenterology, Jinshan Hospital Affiliated to Fudan University, Shanghai, China; ^2^Shanghai Mobio Biomedical Technology Co. Ltd., Shanghai, China

**Keywords:** curcumin, gut microbiota, irritable bowel syndrome, neurotransmitters, constipation

## Abstract

**Introduction:**

Constipation-predominant irritable bowel syndrome (IBS-C) is a functional bowel disease that affects 10–20% of the population worldwide. Curcumin (CUR) is widely used in traditional Chinese medicine to treat IBS, but its mechanism of action needs further investigation.

**Methods:**

In this study, we used mosapride (MOS) as a positive control to evaluate the changes in gut microbiota in IBS-C rat models after treatment with CUR or MOS by analyzing 16S rDNA variation. In addition, we used enzyme immunoassay kits and immunohistochemical analysis to investigate whether CUR or MOS influenced serotonin (5-HT), substance P (SP), and vasoactive intestinal peptide (VIP) levels in the serum and colon of IBS-C rats.

**Results:**

The study showed that rats supplemented with CUR showed significantly increased fecal weight, fecal water content, small intestine transit rate and significantly decreased serum levels of 5-HT, VIP and SP compared to the IBS group (*p* < 0.05). In addition, treatment with CUR changed the relative abundance of *Blautia*, *Sutterella*, *Acetanaerobacterium* and *Ruminococcus*2 in the gut microbiota.

**Discussion:**

This study showed that the efficacy of CUR on IBS-C was possibly by modulating the microbiota and lowering the serum levels of HT, SP, and VIP.

## 1 Introduction

Constipation-predominant irritable bowel syndrome (IBS-C) is a functional bowel disorder characterized by disturbed bowel habits and recurrent abdominal pain often associated with defecation (Defrees and Bailey, 2017; Di Rosa et al., 2023). According to global epidemiological studies, IBS affects approximately 7% of the population in Southeast Asia and the Middle East, while the prevalence—in Southern Europe, Africa, and South America ranges from 15% to 21% (Black and Ford, 2020). In addition, women are more likely to develop IBS-C than men (Hadjivasilis et al., 2019), and some symptoms of IBS-C are more prevalent in female patients and in people between the ages of 20–49 years (Kosako et al., 2018). IBS is becoming more common and is evolving into a chronic recurrent condition due to dietary and lifestyle changes (Enck et al., 2016; Di Rosa et al., 2023). Although IBS does not have a significant mortality rate, it causes long-term symptoms in patients, increases the psychological and economic burden of patients, and significantly limits their quality of life (Zhang et al., 2016; Tack et al., 2019).

The etiologic factors of irritable bowel syndrome are complex, and the mechanism is not yet fully understood. Major risk factors include genetics, diet, gut motor dysfunction, gut microbiota imbalance, alterations in gastrointestinal hormones, and psychological factors that may affect the brain-gut axis. In recent years, the role of the gut microbiota in gut motility disorders has been widely studied and discussed. Previous studies have suggested that an imbalance in the gut microbiota may be involved in the development of IBS-C (Ohkusa et al., 2019; Sun et al., 2019; Wen et al., 2020). Gastrointestinal motility is mainly controlled by the enteric nervous system, which regulates gastrointestinal activity, including neurotransmitters such as 5-hydroxytryptamine (5-HT), substance P (SP), and vasoactive intestinal peptide (VIP; Sun et al., 2023). In addition, a clinical study found that the gut microbiota of patients with IBS-C is significantly different from that of healthy individuals, which may influence gut habits (Parthasarathy et al., 2016). Serotonin signaling also plays an important role in the pathogenesis of IBS. Concentrations of 5-HT have been found to be significantly elevated in patients with IBS compared with controls and are associated with intestinal motility (Smith and Koh, 2017; Xi et al., 2019). Recent studies indicate that the gut microbiota is involved in the biosynthesis of 5-HT, while the concentrations of 5-HT in the gut are directly or indirectly regulated by the gut microbiota (Bhattarai et al., 2017; Agus et al., 2018). Studies have also shown that patients with IBS have abnormal levels of gastrointestinal hormones such as 5-HT, SP and VIP (Sun et al., 2023). Most patients also have long-term emotional problems such as depression, anxiety, and tension, which exacerbate functional disorders such as gastrointestinal motility and secretion via the brain-gut axis and further aggravate the condition of IBS patients.

Curcumin (CUR) is the major active constituent of *Curcuma longa*. Modern studies have demonstrated its potent anti-inflammatory (Kocaadam and Şanlier, 2017), antioxidant (Ahangarpour et al., 2019), and antidepressant (Chang et al., 2016) effects. 5-HT is an important neurotransmitter and is closely related to the regulation of intestinal motility. It has been found that treatment with CUR can modulate 5-HT levels, with treatment with CUR significantly increasing 5-HT levels in the hippocampus, while decreasing 5-HT levels in the colon in IBS models (Yu et al., 2015). SP is a neuropeptide involved in conduction and sensory transmission in the intestine. VIP is a neuropeptide that regulates intestinal smooth muscle relaxation and secretory function (Liu et al., 2015). In addition, CUR has low bioavailability (Cheng et al., 2023), and most of the unmetabolized CUR is excreted in the feces, which means that CUR can interact directly with the gut microbiota. CUR has been shown to positively affect the gut microbiota and modulate microbiota composition and function, further improving gut health (Ng et al., 2018). These studies support the potential therapeutic effect of CUR on IBS-C through modulation of the gut microbiota and neurotransmitters.

Because of the close relationship between the gut microbiota, neurotransmitters, and IBS-C, we hypothesized that treatment with CUR may modulate the gut microbiota and levels of 5-HT, VIP and SP. To test this hypothesis, mosapride (MOS) was used as a positive control for the efficacy on IBS-C in this study, and 16S rDNA variation analysis was used to observe the changes in gut microbiota communities in IBS-C rat models after treatment with CUR.

## 2 Materials and methods

### 2.1 Reagent

CUR, MOS, and 0.5% sodium carboxymethylcellulose used in this study were purchased from Sigma Chemical Co. in the United States.

### 2.2 Animals and experimental design

Twenty-eight specific pathogen free (SPF) male Sprague–Dawley rats weighing 160 to 200 grams and 6 weeks of age were used for this study. They were provided by Shanghai Jishijie Laboratory Animal Co., Ltd. and maintained under standard conditions, including a temperature of 22°C ± 2°C, a relative humidity of 55% ± 5%, and a 12-h light–dark cycle. After 2 weeks of adaptive training, the rats were randomly divided into four groups: normal control group (CTL), IBS model group (IBS), MOS + IBS group (MOS), and CUR + IBS group (CUR). Rats in the IBS model, MOS and CUR groups received 0°C saline (2 mL/day) in the stomach for 2 weeks to induce the IBS-C rat model. Subsequently, rats in the MOS and CUR groups received 2 mL MOS or 0.2 g/kg body weight CUR daily for 2 weeks, whereas rats in the CTL and IBS groups received the corresponding normal saline. Successful model establishment was partially confirmed by the assessment of the physiological characteristics of the rats, including body weight loss, decreased food intake and defecation frequency, dry feces, disheveled hair, and signs of mental fatigue. All rats had free access to food and water throughout the study.

**Figure 1:**
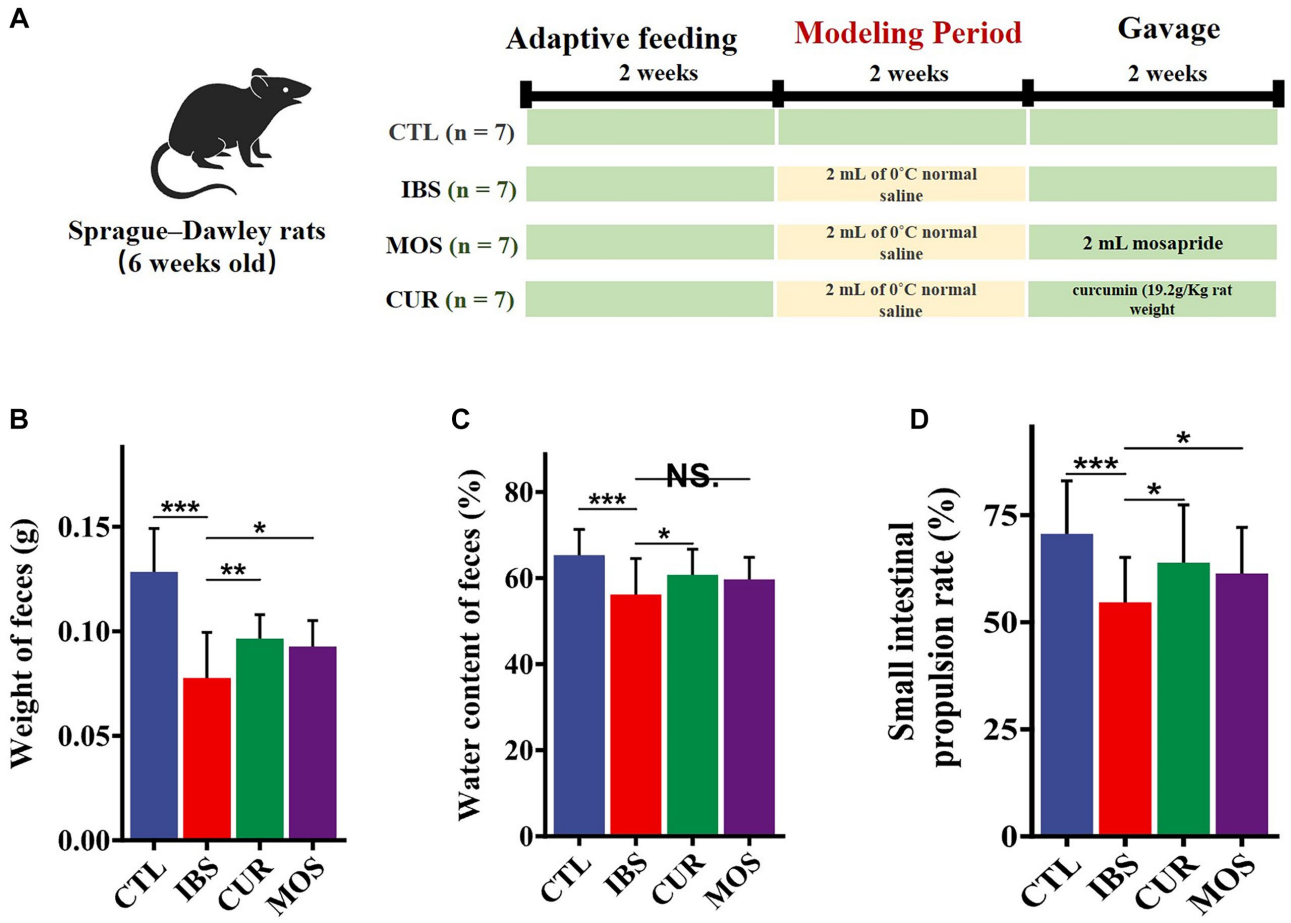
Experimental procedures and the palliative effect of CUR and MOS on constipation in IBS-C rats. Establishment process and intervention methods of IBS-C rat models **(A)**. Effects of CUR and MOS on stool weight **(B)**, stool water content **(C)**, and small intestine transit rate **(D)** in IBS-C rats. Results are indicated as mean ± SD and comparison between groups were made using the Student t test (^*^p < 0.05; ^**^p < 0.01; ^***^p < 0.001; NS, not significant).

Rats experiments were conducted in accordance with the National Institutes of Health Guidelines for the Care and Use of Laboratory Animals. The study procedures were approved by the Laboratory Animal Welfare & Ethics Committee of Shanghai Public Health Clinical Center (2023-A026-01) to minimize pain and avoid harm. The experimental procedure is shown in Figure 1A.

### 2.3 Determination of feces water contents

Each group of rats was placed individually in a cage and observed for a period of 3 h. During this time, the number and weight of excreted feces were recorded for each rat and fecal samples were collected. The fecal samples were then dried in an oven and weighed again. The water content of the feces was calculated using the following formula: % water content = (wet weight of feces (g) – dry weight of feces (g))/wet weight of feces (g) × 100%.

### 2.4 Small intestine transit rate

After a 12-h fast, all rats received 2 mL of 2% India ink and were euthanized 30 min later. Samples of the small intestine were then collected from the pylorus to the cecum. Small intestinal transit rate was calculated according to the following formula: Small intestine transit rate = length facilitated by ink (cm)/total intestine length (cm) × 100%.

### 2.5 Histopathological analysis of rat colon

Colonic tissue samples were collected from each rat 6 cm above the anus and 3 cm long. Fixation was performed with 10% paraformaldehyde, followed by dehydration with ethanol, alcohol soaking in xylene, and finally kerosene embedding. The embedded tissues were cut into 5 μm thick sections and stained with Hematoxylin and Eosin (H&E).

### 2.6 Immunohistochemical analysis

The colon sections were incubated with anti-5-HT (ab6336, Abcam, United States) and anti-VIP (ab30680, Abcam, United States) antibodies (dilution 1:100) for 24 h at 4°C. Subsequently, the immunoreaction was revealed by using HRP-labeled secondary goat anti-rat antibody (ab97057, Abcam, United States, dilution 1:500) for 2 h at room temperature. Then the slides were stained with 3,3′-Diaminobenzidine (DAB) substrate solution, and counterstained with Hematoxylin. The slides were scanned using a microscope (Olympus, Tokyo, Japan). Five high-power fields (×400 magnification) from each slide were randomly selected and analyzed using Image-Pro Plus image analysis software. Integrated option density (IOD) and area value of each image were measured, and then mean density (mean density = IOD/area) was calculated. The mean optical density values were used to plot the relative expression levels of the proteins.

### 2.7 Serum 5-HT, VIP and SP analysis

Rats were anesthetized with 10% chloral hydrate and sacrificed, and blood samples were collected from the jugular vein. The collected blood samples were placed in centrifuge tubes and centrifuged (e.g., 3,000 rpm for 10 min) to separate plasma or serum. After centrifugation, serum was transferred to a new centrifuge tube and stored at −80°C for subsequent experimental analysis. Finally, serum levels of 5-HT, VIP and SP were measured using enzyme immunoassay kits (Shanghai Mobio Biomedical Technology Co., Ltd.) provided by the manufacturer according to the instructions.

### 2.8 DNA extraction and 16S rDNA sequencing analysis of rat feces

Twenty-eight rat feces samples (seven from each group respectively) were collected at week 4 and stored frozen at −80°C until DNA extraction. DNA was extracted from the fecal samples using the QIAamp DNA Stool Mini Kit. The V3–V4 region of 16S rRNA was amplified using primers 341F and 805R (Li et al., 2020). All PCR products were purified using the Axy PrepDNA Gel Extraction Kit (Axygen Biosciences, Union City, CA, United States) and quantified by fluorescence using the FTC-3000TM real-time PCR system. Subsequently, the purified DNA samples were homogenized and sequenced using the Illumina Hiseq PE300 platform (Shanghai Mobio Biomedical Technology Co., Ltd.). This data has been uploaded to a repository, the SRA number is PRJNA1003150.

### 2.9 Bioinformatic analysis and statistical analysis

Reads were spliced using Flash software, and sequence quality was checked using Mothur software (version 1.44.1) to obtain optimized sequences (Schloss et al., 2009). Sequences were then clustered into operational taxonomic units (OTUs) based on 97% similarity. The α-diversity analysis, including ACE, Chao1, Shannon, and Simpson indices, and β-diversity analysis were performed using Mothur software. Based on OTU abundance and distance, R software (version 4.3) was used to visualize bacterial community classification and distribution, e.g., principal coordinate analysis (PCoA). Linear discriminant effect size analysis (LEfSe; Liu et al., 2021) and nonparametric Kruskal-Wallis rank sum test (KW) were used to detect different profiles between groups. Means of each group were compared by one-way comparison (ANOVA), and differences between the 2 groups were determined by Student’s *t*-test. *p-*values less than 0.05 were considered statistically significant.

## 3 Results

### 3.1 Improved effect of CUR and MOS on constipation in IBS-C rats

To verify the effect of CUR and MOS on constipation in IBS-C rats, we measured the water content and weight of the stool of rats. The results showed a significant decrease in fecal pellet weight in the IBS group compared to the control group (*p* < 0.05). In addition, fecal water content was also significantly decreased in the IBS-C group compared to the CTL group (*p* < 0.05). However, rats supplemented with CUR and MOS showed significantly increased fecal weight (Figure 1B) and increased fecal water content (Figure 1C) compared to the IBS group, indicating that CUR and MOS have a good effect on improving constipation.

### 3.2 Small intestinal transit rate

The results of the test of intestinal transit rate showed that IBS-C rats had significantly lower intestinal transit rate compared with normal controls. However, rats supplemented with CUR and MOS showed significantly increased small intestinal transit rates compared with the IBS group (Figure 1D), suggesting that CUR and MOS can improve the peristaltic capacity of the intestine.

### 3.3 Histological analysis of the colon

**Figure 2:**
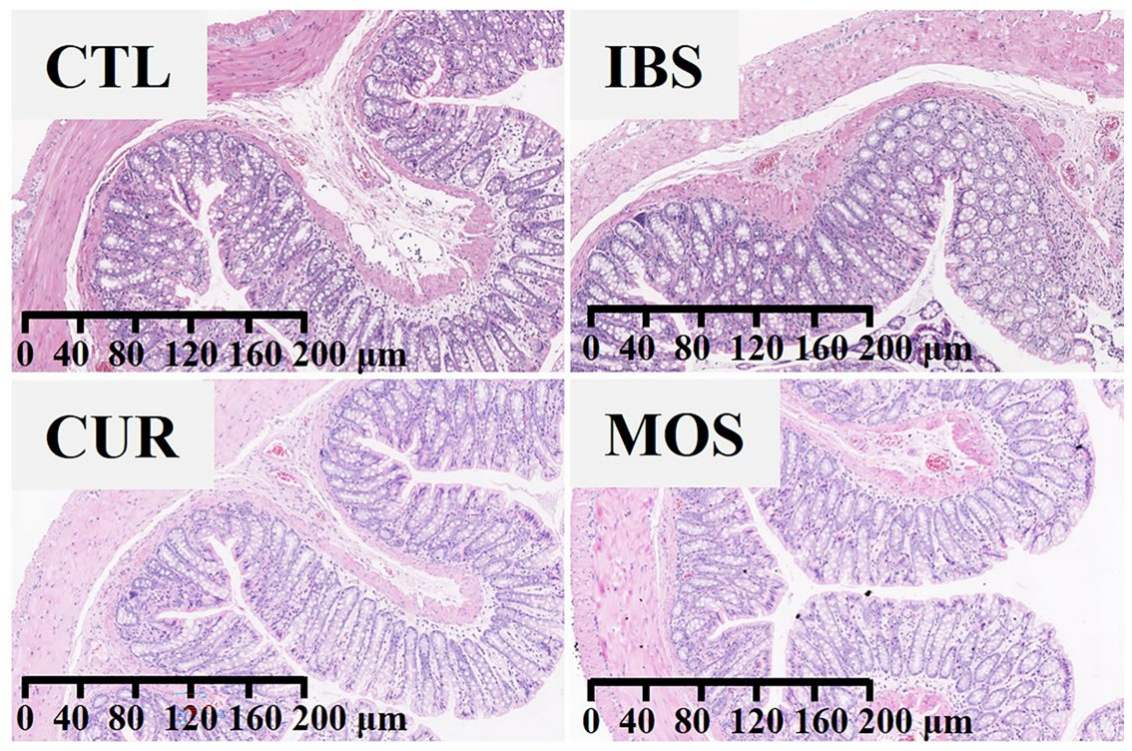
The effect of CUR and MOS on colon tissue using Hematoxylin and Eosin (H&E) staining (viewed with white light).

According to the results in Figure 2, there were no obvious differences in the histological characteristics of the colon among the four groups. The structure of the colon tissue was normal in all groups, with intact colon mucosal epithelium and well-organized glandular arrangement. No congestion, edema, ulceration, inflammatory cell infiltration, or other pathologic changes were observed in any group.

### 3.4 Results of histochemical analysis of the colon

**Figure 3:**
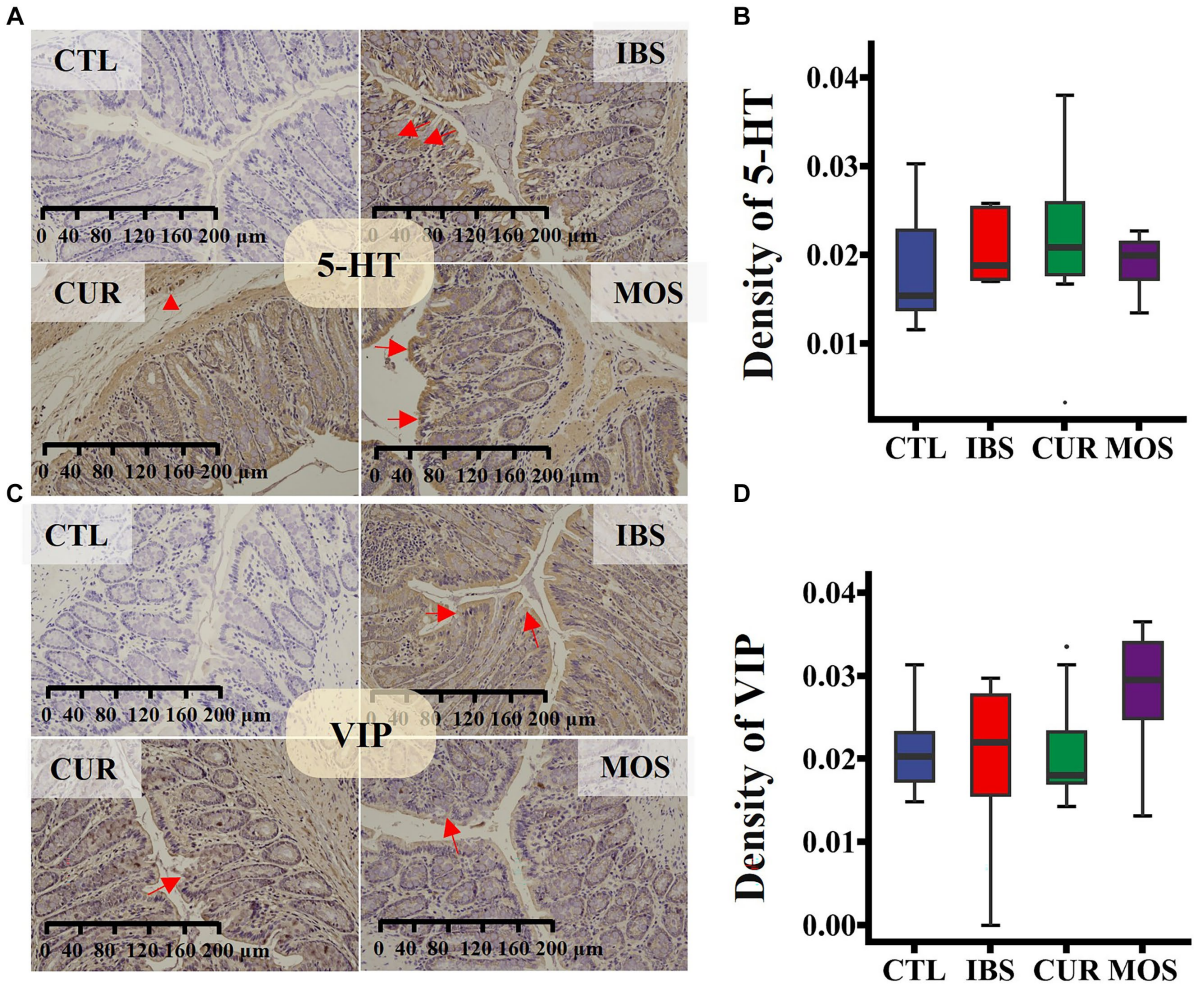
Immunohistochemical staining for 5-HT **(A,B)** and VIP **(C,D)** in colon tissue. Specific proteins are brown after 3,3′-Diaminobenzidine staining, and cell nucleus are blue after Hematoxylin counterstaining (viewed with white light). In panels **(A,C)**, red arrows point to the positive cytoplasmic reaction, red triangle shows strong positive staining in enteric neurocyte. In plots **(B,D)**, the transverse lines in boxplot mean the median.

The immunohistochemical results showed that the immunoreactivity of 5 HT and VIP was mainly concentrated in the colonic mucosa, circular muscle, and intestinal muscle bundles of rats (Figures 3A,C). Remarkably, we did not detect significant changes in the expression of 5-HT and VIP in rats after treatment with CUR or MOS (Figures 3B,D).

### 3.5 Effects of CUR and MOS on serum levels of 5-HT, VIP and SP

**Figure 4:**
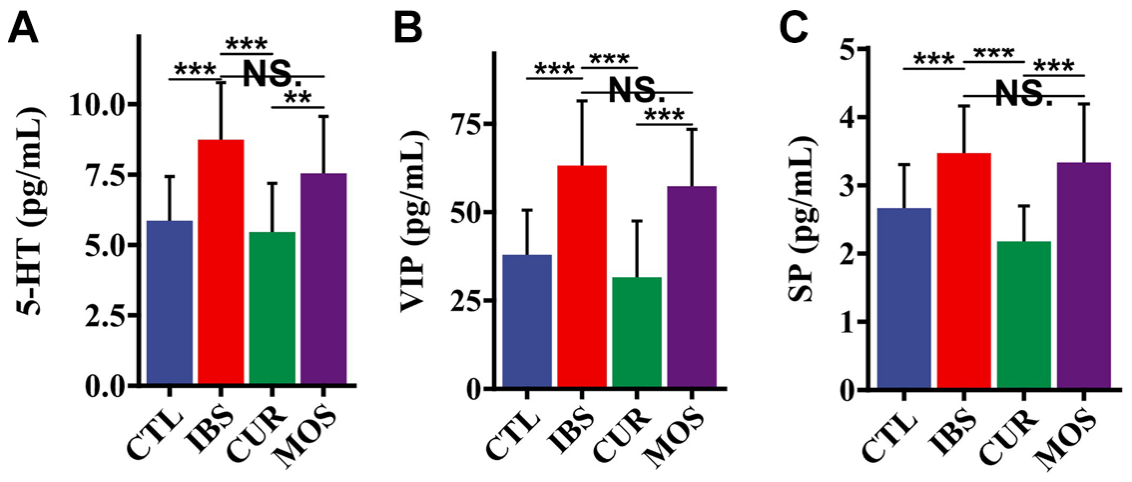
Effect of CUR and MOS on serum levels of 5-HT **(A)** VIP **(B)** and SP **(C)** in IBS-C rats. 5-HT, 5-hydroxytryptamine; VIP, vasoactive intestinal peptide; SP, substance P. Results are indicated as mean  ±  SD and comparison between groups were made using the Student *t* test (^**^p <  0.01, ^***^p <  0.001, NS, not significant).

Since we did not find any significant differences in the concentrations of HT and VIP between the different groups in the colon tissue, we further investigated the serum concentrations of HT, VIP and SP. The results showed that the serum levels of 5-HT, VIP and SP in the IBS group were significantly higher than those in the CTL group (*p* < 0.05). However, treatment with CUR significantly decreased the serum levels of 5-HT, VIP and SP (Figure 4). Notably, the serum levels of HT, VIP and SP were not significantly different in the MOS group compared to the IBS group (*p* > 0.05; Figure 4). These results suggest that CUR significantly decreases serum levels of neurotransmitters compared to MOS.

### 3.6 Effects of CUR and MOS on the intestinal microbiota in IBS-C rats

**Figure 5:**
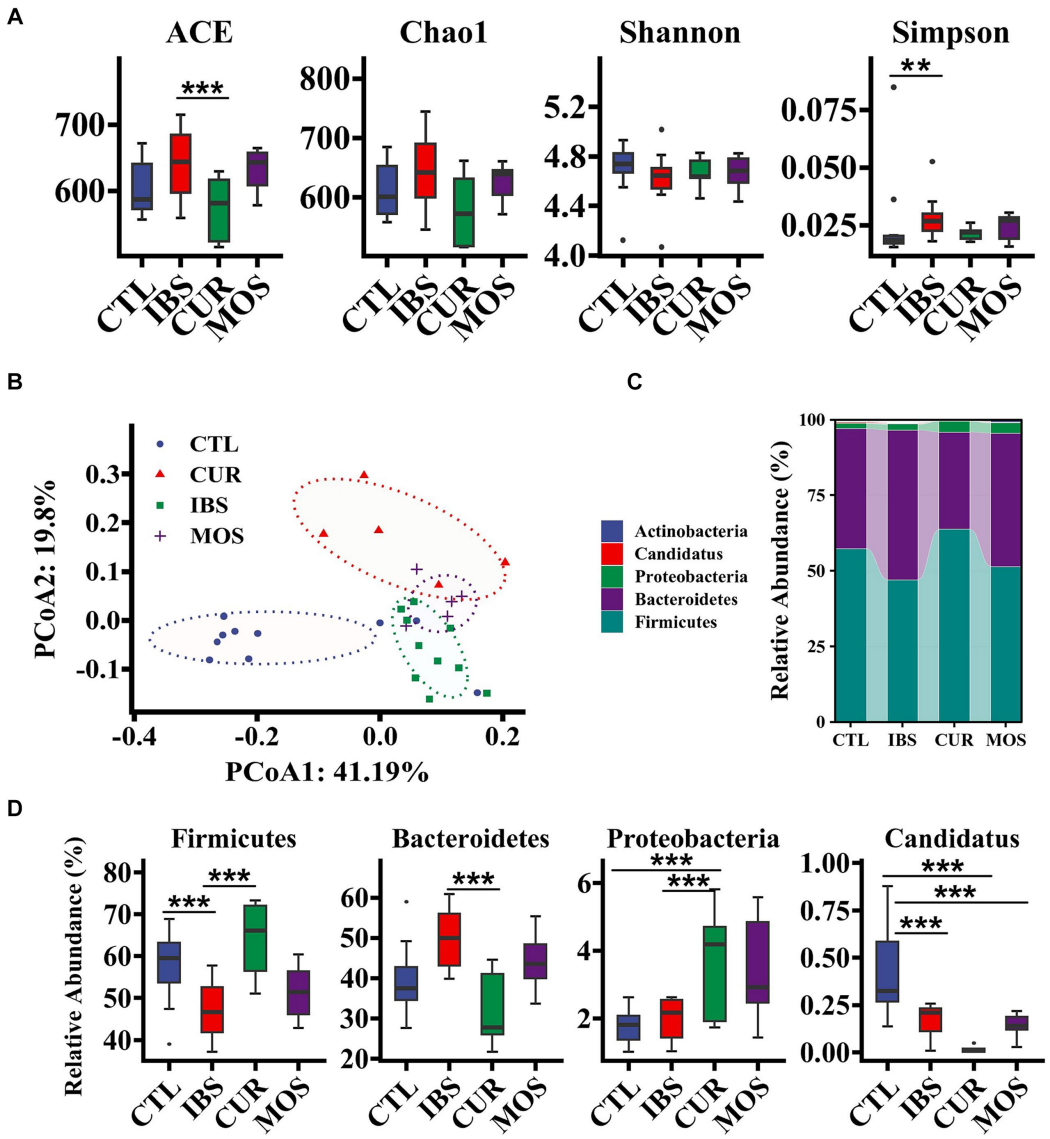
Effect of treatment with CUR or MOS on gut microbiota in IBS-C rats. Panels **(A,B)** show the effect of treatment with CUR or MOS on the alpha and beta diversity of the gut microbiota in IBS-C rats. Panels **(C,D)** show the effects of treatment with CUR or MOS on the relative abundance of gut microbiota at the phylum level in IBS-C rats. The transverse lines in boxplot mean the median. ^*^p < 0.05, ^**^p < 0.01, and ^***^p < 0.001.

We further investigated the effects of CUR and MOS on the intestinal microbiota of IBS-C rats using 16S rRNA sequencing. The gut microbiota of rats in the IBS group showed increased richness (ACE and Chao1 index) and decreased diversity (Shannon index) compared with the CTL group (Figure 5A). However, both the richness and diversity of the gut microbiota improved in the IBS-C rats after treatment with CUR and MOS. We observed a significant decrease in the ACE index in the CUR group (*p* < 0.05).

Then, we performed multivariate analysis using principal coordinate analysis (PCoA) based on Unifrac distance to analyze the global structural changes in the gut microbiota. The PCoA score showed that the gut microbiota in the IBS group had significant structural changes on the first principal component (PCoA1) and the second principal component (PCoA2) compared with the CTL group (Figure 5B). However, treatment with CUR and MOS significantly reversed this change, with CUR having a more dramatic effect on the gut microbiota (Figure 5B). Thus, the structure of the gut microbiota was altered in IBS-C rats, and treatment with CUR had a greater effect on the gut microbiota.

At the phylum level, the predominant components of the microbiota were Firmicutes and Bacteroidetes, followed by Proteobacteria, which had relative abundances above 99. 9% (Figure 5C). Compared with the CTL group, the IBS group showed a significant decrease in the relative abundance of Firmicutes and Candidatus, whereas Bacteroidetes showed a significant increase. This trend reversed significantly after the intervention of CUR. The relative frequency of Firmicutes was significantly increased in the CUR group compared to the IBS group, while the frequency of Bacteroidetes was significantly decreased. In addition, MOS showed a similar trend, but no significant difference compared with the IBS group. Of note, both the CUR and MOS interventions resulted in an increase in Proteobacteria abundance (Figure 5D).

### 3.7 LEfSe analysis and correlation analysis

**Figure 6:**
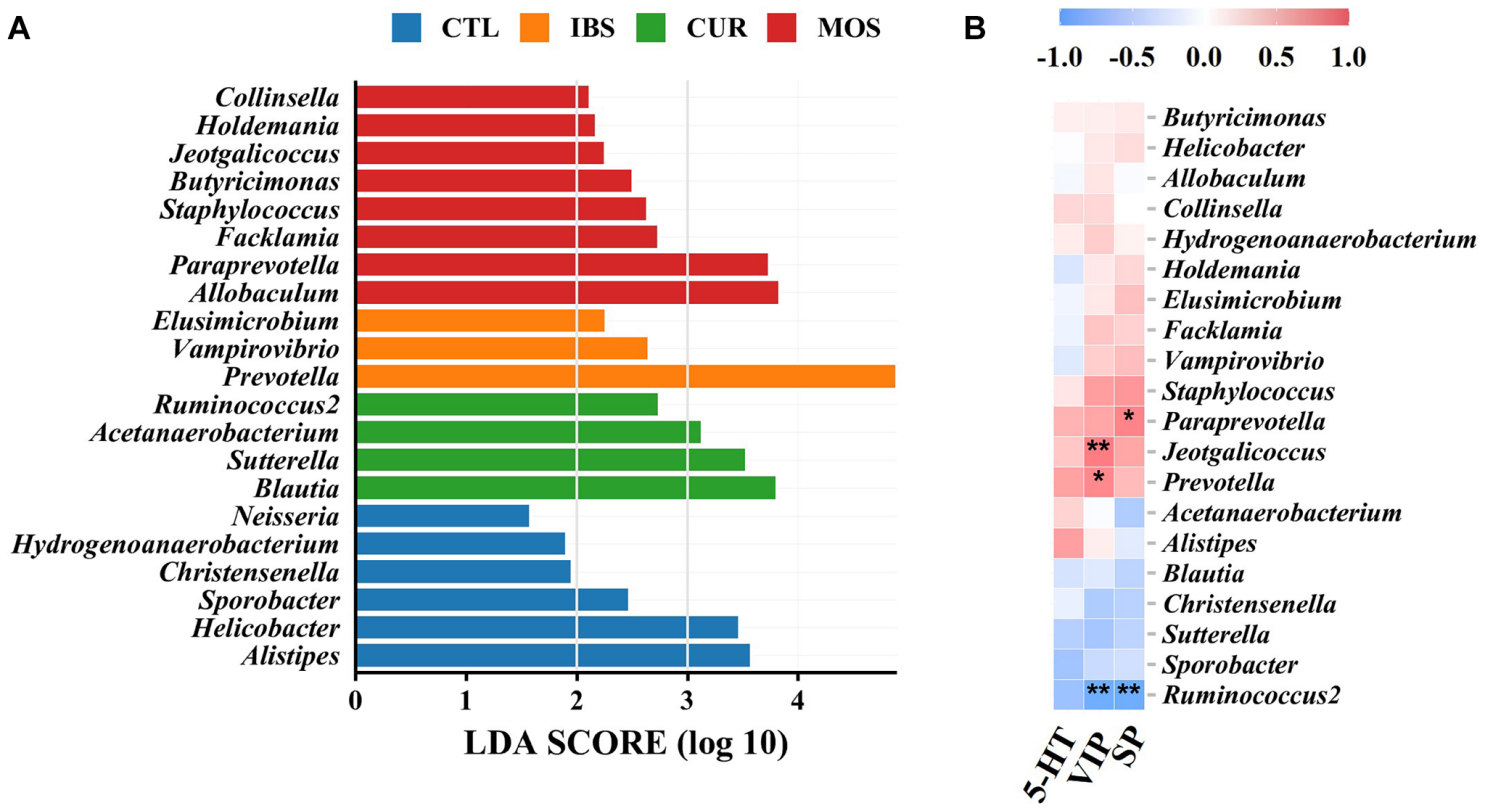
LEfSe analysis and correlation analysis. **(A)** LEfSe analysis. **(B)** Heatmap of Pearson correlation analysis, blue-white-red color: negative correlation-no correlation-positive correlation. ^**^p < 0.01, ^*^p < 0.05. 5-HT, 5-hydroxytryptamine; VIP, vasoactive intestinal peptide; SP, substance P.

LEfSe analysis showed that *Alistipes*, *Helicobacter*, *Sporobacter*, *Christensenella*, *Hydrogenoanaerobacterium* and *Neisseria* were enriched in the CTL group. *Prevotella*, *Vampirovibrio* and *Elusimicrobium* were increased in the intestine of rats of the IBS-C model, while *Blautia*, *Sutterella*, *Acetanaerobacterium* and *Ruminococcus*2 were enriched according to CUR. In contrast, treatment with MOS resulted in increased abundance of *Allobaculum*, *Paraprevotella*, *Facklamia*, *Staphylococcus*, *Butyricimonas*, *Jeotgalicoccus*, *Holdemania*, and *Collinsella* (Figure 6A). In addition, correlation analysis showed that the enrichment of *Ruminococcus*2 in the CUR group was negatively correlated with VIP and SP, while the enrichment of *Prevotella* in the IBS group and *Jeotgalicoccus* in the MOS group were positively correlated with VIP (Figure 6B). The enrichment of *Paraprevotella* in the MOS group was positively associated with SP.

## 4 Discussion

In this study, we developed a rat model of IBS with 0°C saline. The results showed that after 2 weeks of modeling, the number of fecal pellets and water content were significantly lower in the IBS group than in the CTL group, indicating that the rats in the IBS group developed constipation symptoms. In addition, H&E staining of the colon tissue showed no significant pathological changes in the structure of the colon tissue of the IBS rats compared with the CTL group, which is consistent with previous findings that IBS-C is not associated with an inflammatory response and damage to the colon epithelial cells (Peters et al., 2017).

5-HT, VIP and SP are ubiquitous peptides in the central and enteric nervous systems that are closely related to the development of IBS and play an important regulatory role in visceral sensation and movement (Liu et al., 2015; Liang et al., 2016). VIP may relax gastrointestinal smooth muscle and inhibit gastrointestinal motility, while SP may enhance gastrointestinal peristalsis, promote gastrointestinal smooth muscle contraction, and support group movement of the colon (Tang et al., 2022; Fan et al., 2023). Visceral hyperalgesia in patients with IBS is associated with an increase in the content of 5-HT in their peripheral tissues (Guo et al., 2022). In this study, immunohistochemical analysis showed that the levels of 5-HT, SP, and VIP in colon tissues were not significantly different between the different groups. However, the serum levels of 5-HT, SP and VIP were significantly increased in rats with irritable bowel syndrome. In contrast, the levels of 5-HT, SP and VIP were significantly decreased in the serum of CUR and MOS rats. These neurotransmitters are closely associated with symptoms such as constipation, bloating, and abdominal pain. In a 30-day randomized trial, in subjects with IBS, abdominal bloating can be successfully reduced with a supplementation with CUR (Giacosa et al., 2022). Previous studies have shown that CUR has a significant effect on reducing the levels of 5-HT in serum and colon (Yu et al., 2019). The intestine is the source of most serotonin in the human body, which is predominantly located in the endocrine cells of the intestinal epithelium (Ge et al., 2018).

In this study, we also investigated changes in the composition of the gut microbiota using 16S rRNA sequencing. Consistent with previous results, PCoA analysis showed distinct distances between groups, suggesting that the diversity of the gut microbiota in IBS-C rats and CUR /MOS-treated rats differs from that of normal rats. In the present study, we observed increased abundance of Bacteroidetes in the IBS rat model, which is consistent with previous studies and shows that Bacteroidetes may be a potentially harmful microbiota in patients with IBS (Ponnusamy et al., 2011; Wang et al., 2018; Pittayanon et al., 2019). In addition, we found high relative abundances of *Prevotella*, *Vampirovibrio*, and *Elusimicrobium* in rats with IBS-C. Previous studies have shown that *Prevotella* is strongly associated with a high risk of IBS-D and can interact with other members of the gut microbiota and cause visceral allergic reactions that further exacerbate IBS symptoms (Kovatcheva-Datchary et al., 2015; Su et al., 2018). In addition, *Prevotella* is thought to be associated with gut permeability and proinflammatory function (Larsen, 2017; Guerreiro et al., 2018). Some studies have shown that *Allobaculum*, a bacterium that produces short-chain fatty acids, can alleviate the symptoms of irritable bowel patients by regulating intestinal transit time (Camilleri, 2015). We observed a significant increase in the frequency of *Allobaculum* in rats from the MOS group. In addition, the abundance of *Ruminococcus*2 decreased in CUR group. Interestingly, these changes in the gut microbiota associated with IBS were partially reversed by treatment with CUR, which has been observed in other disease models. Some studies have shown that the gut microbiota was reversed by CUR, including Bacteroidetes, a kind of microbiota potentially harmful in patients with IBS (Huang et al., 2021). *Ruminococcus*2 is considered a protective microbiota in IBS patients as it maintains the integrity of the intestinal mucosa and reduces IBS symptoms (Rivière et al., 2016). In conclusion, the results of this study show that CUR was able to lower the levels of HT, VIP and SP and modulate the gut microbiota in rats with IBS-C. However, it is worth noting that there is significant heterogeneity among the studies and even with modern technologies, no uniform characteristics of IBS-related gut microbiota has been identified. Further studies are needed to investigate the regulatory mechanism of CUR on gut microbiota in IBS-C rats.

In this study, the effects of CUR and MOS on rats with IBS-C were comprehensively investigated by histological analysis, immunohistochemistry, and 16S rRNA sequencing, and provided important clues for studying the microbiological mechanism of IBS-C. Determination of neurotransmitter levels in serum also improves our understanding of the role of neurotransmitters in disease development and provides clues for therapeutic strategies. However, this study also has several shortcomings, including small sample size, limitations of animal models, and lack of validation of clinical data. To improve the reliability and generalizability of the study, we need to perform more clinical validations and a more comprehensive experimental design. Overall, these results provide valuable information for the treatment and microbiological study of IBS-C and provide a foundation for future research, but more in-depth studies are needed to confirm and refine these results. Future work should more comprehensively investigate neurotransmitter changes and their interactions with the gut microbiota to better understand the role of the brain-gut axis in the treatment of IBS-C (Sun et al., 2023).

## 5 Conclusion

The results of this study show that CUR is able to decrease the levels of HT, VIP and SP and regulate the gut microbiota in rats with IBS-C, and then exert the therapeutic effect on IBS-C. The results suggest that CUR may represent a treatment option for IBS-C through modulation of the gut microbiota and relevant neurotransmitters. Our results support the possibility of using CUR to treat IBS-C patients.

## Data availability statement

The data presented in the study are deposited in the National Center for Biotechnology Information database, accession number PRJNA1003150.

## Ethics statement

The animal study was approved by Shanghai Public Health Clinical Center Laboratory Animal Welfare and Ethics. The study was conducted in accordance with the local legislation and institutional requirements.

## Author contributions

XT: Writing – original draft, Writing – review & editing. HR: Writing – review & editing, Data curation. SB: Writing – review & editing, Funding acquisition, Investigation, Methodology, Project administration, Resources.
